# Enhancement of Tetravalent Immune Responses to Highly Conserved Epitopes of a Dengue Peptide Vaccine Conjugated to Polystyrene Nanoparticles

**DOI:** 10.3390/vaccines8030417

**Published:** 2020-07-25

**Authors:** Yanqi Chan, Seyed Davoud Jazayeri, Babu Ramanathan, Chit Laa Poh

**Affiliations:** 1 Centre for Virus and Vaccine Research, School of Science and Technology, Sunway University, Subang Jaya 47500, Malaysia; 17029422@imail.sunway.edu.my (Y.C.); sjazayeri@sunway.edu.my (S.D.J.); 2 Department of Biological Sciences, School of Science and Technology, Sunway University, Subang Jaya 47500, Malaysia; babur@sunway.edu.my

**Keywords:** dengue vaccine, peptide vaccine, polystyrene nanoparticles (PSNPs), humoral immune responses

## Abstract

Vaccination remains the major approach to the prevention of dengue. Since the only licensed live attenuated vaccine (LAV) lacked efficacy against all four serotypes, other vaccine platforms, such as synthetic peptide vaccines, should be explored. In this study, four multi-epitope peptides (P1–P4) were designed by linking a universal T-helper epitope (PADRE or TpD) to the highly conserved CD8 T cell epitope and B cell epitope (B1 or B2) against all four DENV serotypes. The multi-epitope peptides were conjugated to polystyrene nanoparticles (PSNPs) and four nanovaccines (NP1–NP4) were constructed. Mice immunized with NP1–NP4 elicited significantly higher titers of IgG and neutralizing antibodies when compared to immunization with naked P1–P4. The immune responses in mice immunized with peptide vaccines were compared with nanovaccines using ELISA, ELISPOT, and a neutralization test based on FRNT_50_. Among the four conjugated peptide nanovaccines, NP3 comprising the TpD T-helper epitope linked to the highly conserved B1 epitope derived from the E protein was able to elicit significant levels of IFN-γ and neutralizing antibodies to all four dengue serotypes. NP3 is a promising tetravalent synthetic peptide vaccine, but the selection of a more effective CD8^+^ T cell epitope and adjuvants to further improve the immunogenicity is warranted.

## 1. Introduction

Dengue virus is a mosquito-borne flavivirus with four serotypes (DENV 1–4) and is responsible for putting approximately one-third of the world’s population at risk of dengue infection. There are 390 million infections annually, 96% of which are symptomatic. Severe dengue infections can also lead to dengue hemorrhagic fever (DHF) or dengue shock syndrome (DSS), which is potentially deadly due to plasma leakage, severe bleeding, and organ impairment [1]. DENV is currently endemic in more than 100 countries worldwide and Southeast Asia is one of the most seriously affected regions. In Malaysia, a total of 127,407 DENV cases and 176 deaths were reported in 2019.

Infection with any of the four DENV serotypes confers long-term immunity against the primary (1°) infecting serotype, but only provides transient cross-protection against the remaining DENV serotypes. Severe dengue is more often associated with sequential heterotypic dengue infections. Individuals with prior infection with one serotype may develop life-threatening DENV disease upon exposure to secondary (2°) infection with a heterologous serotype [2]. There are two theories that can explain the increase in severity seen in secondary infections: antibody-dependent enhancement (ADE), which is the humoral response to 2° heterologous dengue infection [3,4], and the equivalent aberrant response in cellular immunity known as the original antigenic sin/Hoskins effect [5,6]. The enhancement of disease upon 2° infection and the need to protect against four diverse serotypes set a high target for the development of an efficacious dengue vaccine.

Vaccination remains the most effective preventive measure, but vaccine development has been slow as immunization may predispose seronegative vaccinees to severe dengue infections. The development of DENV vaccines has been in progress for over 30 years, but no safe and effective vaccines have been produced which could induce protective immunity against all four serotypes of DENV. A number of dengue vaccine candidates are currently under development, such as the live attenuated vaccines (LAVs) [7,8], inactivated vaccines [9], virus-like particle-based vaccines [10], and subunit vaccines [11].

DENGVAXIA^®^, a tetravalent chimeric LAV, was the first dengue vaccine licensed for use in several endemic countries. However, in naïve subjects, there is a potential risk involved with subsequent infections and the vaccine should only be administered to seropositive subjects [12]. For seronegative subjects, the vaccination is treated as the 1° infection, which sensitizes the vaccinees to developing more severe dengue disease upon secondary dengue infections [12]. The main mode of immune response to Dengvaxia^®^ appears to be humoral by eliciting neutralizing antibodies. The imbalanced antibody titers or waning of protective antibody titers over time could also play a part in increasing the risk of developing severe dengue in seronegative subjects that are vaccinated with DENGVAXIA^®^ [13]. The key advantage of LAV is its ability to mimic natural virus infection and trigger robust humoral- and cellular-mediated immune responses. However, LAVs come with the risk of reversion to wild-type virulence, rendering them unsuitable for individuals who are immunocompromised.

Synthetic peptide vaccines offer substantial advantages over LAV due to their higher safety profiles, as they can provide long-term vaccine stability with no risk of genetic reversion. Synthetic peptide vaccines (or “designer” vaccines) are being developed as prophylactic vaccines for the prevention of viral infections [14]. In an adaptive immunological response against foreign antigens, only short segments of the antigens (termed epitopes) are recognized by the B and T cell receptors [15]. This type of vaccine consists of selected epitope(s) of the antigenic protein, thus enabling the induction of highly targeted immune responses. Compared to conventional vaccines, peptide synthesis is reproducible, non-infectious, and nonreplicating [16]. However, despite its high safety profile compared to LAVs, synthetic peptide vaccines are generally poorly immunogenic. Moreover, they are susceptible to enzymatic degradation, making them unstable in vivo [17]. These limitations can be overcome by using several strategies, such as the addition of T-helper epitopes, increasing the peptide length, using nanoparticles as the vaccine delivery vehicle, or a combination of several approaches [18].

The pan HLA-DR-binding epitope (PADRE) is a universal synthetic peptide that activates CD4 T cells and is frequently employed for synthetic or recombinant vaccine development [19]. TpD is a chimeric universal T-helper epitope consisting of epitopes from the tetanus toxoid and diphtheria toxoid separated by an internal cathepsin cleavage site. Immunization with TpD was found to produce a promising antibody response and was capable of promoting a long-term CD4 immune response in different animal models and humans [20]. These two different universal T-helpers can be incorporated in the synthetic peptide to enhance the immunogenicity of the peptide vaccine [20,21].

Since peptide antigens used in vaccines are poorly immunogenic, particulate carriers such as nanoparticles have been frequently employed for their adjuvanting properties and delivery [18]. Several studies in animal models have shown that when the target antigens of choice were covalently conjugated to inert nanoparticles, such as biocompatible carboxylated polystyrene nanoparticles (PSNPs), they were capable of inducing potent CD8 and CD4 T cell responses, as well as antibody responses, in vivo following immunization [22,23]. In this study, we designed four conserved synthetic peptide-based vaccines consisting of either of the two different universal T-helper epitopes PADRE or TpD, producing a CD8 T cell epitope derived from the DENV NS5 protein, and either of the two different B cell epitopes derived from the Envelope (B1) or the NS4A (B2) protein. The B cell epitopes B1 and B2 were identified using *in silico* predictions and were reported to be highly conserved in all DENV serotypes, making them good candidate targets for the development of a tetravalent synthetic peptide vaccine. To improve the immunogenicity of these four peptide constructs, we evaluated the conjugation of the peptides to carboxylated PSNPs using covalent conjugations and compared the magnitudes of the immune responses elicited by their corresponding peptides. PSNPs were effective as adjuvants for significantly increasing the immunogenicity of the multi-epitope peptide vaccines. Mice immunized with peptides conjugated to the PSNPs were able to induce high levels of IgG and significant neutralizing antibody titers to all four DENV serotypes compared to mice immunized with peptides alone.

## 2. Materials and Methods

### 2.1. Cell Lines and Viruses

Vero cells (African green monkey kidney cell line, ATCC^®^, CCL-81TM) were purchased from the American Type Culture Collection (ATCC) (Rockville, MD, USA). The cells were maintained in Dulbecco’s Modified Eagle’s Medium (DMEM) (Gibco, Boston, MA, USA) supplemented with 10% fetal bovine serum (FBS) (Gibco, Boston, MA, USA) and 1% penicillin and streptomycin (Pen-Strep) (Nacalai Tesque, Japan) at 37 °C in the presence of 5% CO_2_ in a 95% humidified incubator (Thermo Fisher Scientific, Waltham, MA, USA). DENV strains (DENV prototypes DENV1 (Hawaii), DENV2 New Guinea C (NGC), DENV3 (H87), and DENV4 (H241)) were grown in confluent monolayers of Vero cells in DMEM supplemented with 10% FBS and 1% Pen-Strep at 37 °C in the presence of 5% CO_2_ in a 95% humidified incubator. All DENV strains were propagated and maintained in DMEM with 2% FBS. The virus strains were stored at −80 °C in the freezer (Eppendorf, Germany) for use in future experiments.

### 2.2. Design and Synthesis of Peptides

**Figure 1:**
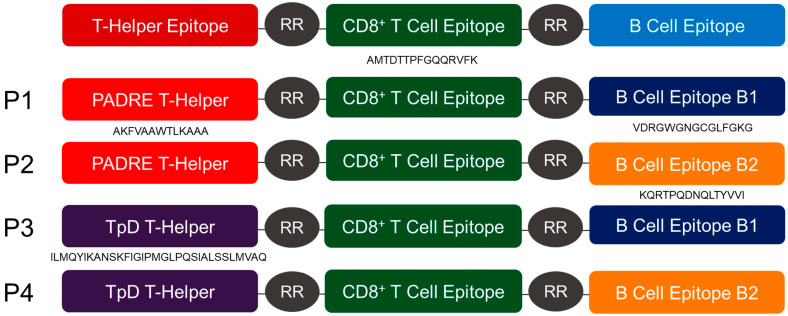
A schematic diagram of the four peptide constructs (P1–P4).

Four multi-epitope peptides were constructed using two different B cell epitopes. The B1 epitope (VDRGWGNGCGLFGKG) was derived from the DENV E protein domain II and identified by Muthusamy et al. (2016) using *in silico* prediction [24], whilst the B2 epitope (KQRTPQDNQLTYVVI) was derived from the NS4A protein and identified *in silico* by Verma et al. (2019) [25]. In addition, two different universal T-helper epitopes were incorporated, which were the artificial pan-DR binding epitope known as PADRE (AKFVAAWTLKAAA) [26] and the chimeric MHC class II epitope, TpD (ILMQYIKANSKFIGIPMGLPQSIALSSLMVAQ), comprising epitopes that were derived from tetanus and diphtheria toxoids. All four multi-epitope peptides shared one common CD8 cytotoxic T cell epitope (AMTDTTPFGQQRVFK) that was derived from the NS5 protein and identified by Shi et al. (2015) using an immunoinformatic approach [27]. Each epitope of the four peptide vaccine constructs (P1–P4) was linked with two “arginine” residues (RR). The R residues were introduced as a protease-sensitive linker, such that once the vaccine was internalized by dendritic cells, intracellular proteases would cleave at the RR bipeptide and separate the epitopes, thus enhancing the processing and presentation of the epitopes [28]. The sequence of Peptide 1 (P1) is AKFVAAWTLKAAA**RR**AMTDTTPFGQQRVFK**RR**VDRGWGNGCGLFGKG, Peptide 2 (P2) is AKFVAAWTLKAAA**RR**AMTDTTPFGQQRVFK**RR**KQRTPQDNQLTYVVI, Peptide 3 (P3) is ILMQYIKANSKFIGIPMGLPQSIALSSLMVAQ**RR**AMTDTTPFGQQRVFK**RR**VDRGWGNGCGLFGKG, and Peptide 4 (P4) is ILMQYIKANSKFIGIPMGLPQSIALSSLMVAQ**RR**AMTDTTPFGQQRV-FK**RR**KQRTPQDNQLTYVVI (Figure 1). The peptides present in the vaccine constructs were synthesized by Mimotopes Pty Ltd. (Melbourne, Victoria, Australia).

### 2.3. Conjugation of Synthetic Peptides to Carboxylated PSNPs

The conjugation of dengue synthetic peptide antigens to PSNPs was based on the method described by Wilson et al. (2015) [23], with slight modifications. Carboxylated PSNPs (Polysciences Inc., Warrington, PA, USA) of 50 nm at a final concentration of 1% solids were pre-activated in a mixture containing 2-*N*-morpholino-ethanesulfonic acid (MES) (50 mM final) (Sigma-Aldrich, St. Louis, MO, USA), 1-ethyl-3-(3-dimethylaminopropyl) carbodiimide hydrochloride (EDAC) (4 mg/mL, final concentration) (Sigma-Aldrich, St Louis, MO, USA), *N*-hydrosulfosuccinimide (Sulfo-NHS) (50 mM, final concentration) (Thermo Fisher Scientific, Waltham, MA, USA), and endotoxin-free water (Thermo Fisher Scientific, Waltham, MA, USA). The pH of MES buffer used was optimized for each peptide antigen (50 mM final, pH = 6.5 for peptide 1 (P1) and peptide 2 (P2), pH = 7.5 for peptide 3 (P3), and peptide 4 (P4)) (Sigma-Aldrich, St Louis, MO, USA). The pre-activation mix was incubated for 1 h on a shaker (IKA, China) at room temperature (RT) and then dialyzed in a 10 K molecular weight cut-off (MWCO) membrane (SnakeSkin™ Dialysis Tubing, Thermo Fisher Scientific, Waltham, MA, USA) against PBS (pH 7.5) at 4 °C overnight. The excess activation agents (EDAC and Sulfo-NHS) were removed from the pre-activation mix following overnight dialysis. The pre-activated PSNP was mixed with the peptide antigens and incubated for 2 h. The conjugation reactions were then quenched by adding excess glycine (7 mg/mL final) and the mixture was further incubated for 30 min. The conjugation mix was then dialyzed in a 10 K MWCO membrane against PBS (pH 7.2) at 4 °C overnight. The dialyzed nanovaccine conjugates were assayed for the conjugation efficiency, size, and nanoparticle recovery.

### 2.4. Determination of the Conjugation Efficiency

The conjugation efficiency of peptides to PSNPs was determined by checking the presence of unconjugated peptides in the supernatant from the conjugation mixture after ultracentrifugation (Type TLA-100 rotor, Beckman Coulter, Fullerton, CA, USA) at 200,000× *g* for 30 min at 4 °C. Peptides in the supernatant were detected by the Bicinchoninic acid (BCA) assay (Micro BCA™ protein assay, Thermo Fisher Scientific, Waltham, MA, USA), following the manufacturer’s instructions.

### 2.5. Determination of the Size of Nanovaccines

The size of the nanovaccines in the formulations was measured using a dynamic light scattering (DLS) instrument (Zetasizer, Malvern Instruments Ltd., Worcestershire, UK). The final conjugation mixture (5–10 µL/each) was diluted in 800 µL of PBS and loaded into a disposable capillary cell (Malvern Instruments Ltd., Worcestershire, UK). The diffusion of particles moving under Brownian motion was measured by the Zetasizer and converted to the particle size through the Stokes–Einstein relationship. After inputting the particle reflective index and the buffer system used (distilled water), the particle size was then calculated by the Zetasizer software (version 7.12).

### 2.6. Determination of Nanoparticle Recovery

The concentration of nanoparticles in suspension was monitored throughout the conjugation process using a Biodrop µLITE spectrophotometer. At each stage, the absorbance of the particle suspension at 248 nm was compared against that of the original particle suspension (before the addition of the peptide antigen). The background absorbance of the particle suspension was collected by ultracentrifugation and was subtracted from the whole particle suspension to give an accurate measurement, as the residual Sulfo-NHS in the conjugation mix could also absorb strongly at the same wavelength. The recovery of nanoparticles was calculated using the following formula:

$$\frac{\left(\mathrm{Particle}\;\mathrm{suspension}\;\mathrm{after}\;final\;\mathrm{dialysis}\;\mathrm{absorbance}\;\mathrm{at}\;248\;\mathrm{nm}-\mathrm{Background}\;\mathrm{absorbance}\right)}{\left(\mathrm{Particle}\;\mathrm{suspension}\;\mathrm{after}\;first\;\mathrm{dialysis}\;\mathrm{absorbance}\;\mathrm{at}\;248\;\mathrm{nm}-\mathrm{Background}\;\mathrm{absorbance}\right)}\times 100.$$

### 2.7. Transmission Electron Microscopy (TEM) Analysis

In addition to using the BCA™ protein assay, the conjugation of peptides to the carboxylated PSNPs was also analyzed via LEO-Libra 120 TEM (Carl Zeiss Ltd., Oberkochen, Germany) through the TEM service provided by the University of Malaya. The nanovaccine formulations (10 μL) were spotted directly onto formvard-coated copper TEM grids (400 square mesh) and allowed to stand for 3 min. The excess solution was blotted with filtered paper, and then stained with 1% (*w/v*) phosphotungstic acid (PTA) for 1 min and allowed to air dry. The specimens were kept in a desiccator for 3 days to allow them to dry. Images displaying PSNPs, peptides, and nanovaccines (NP1-NP4) were obtained by using LEO-Libra 120 TEM operated at an accelerating voltage of 120 kV.

### 2.8. Mice and Immunization

All animal procedures were performed in accordance with protocols approved by the Monash Animal Research Platform (MARP) Animal Ethics Committee (AEC), Monash University, Malaysia (AEC approval no.: MARP/2017/058) and Sunway University Research Ethics Committee (Approval code: SUREC 2017/050). Animals were purchased from Monash Animal Services (MAS). Ten groups, each comprising five female BALB/c mice (4–6 weeks old), were immunized subcutaneously below the loose skin at the back of the neck with 100 µL of each vaccine formulation on days 0, 14, and 28. Sera were collected on days 0, 14, 28, and 42 from the immunized mice and mice from the control group. The mice were sacrificed on day 42, the blood was collected by cardiac puncture, and the spleens were harvested.

### 2.9. Enzyme Linked Immunosorbent Assay (ELISA)

Briefly, each well of an Immulon 2HB (high binding) 96-well flat bottom microtiter plate (Fisher Scientific, Pittsburgh, PA, USA) was coated with 100 µL of 10 µg/mL P1–P4 peptides in carbonate-bicarbonate buffer (pH 9.6) and left overnight at 4 °C. The non-specific antibody binding was blocked by incubation with 1% (*w/v*) bovine serum albumin (BSA) in PBS buffer (BSA_10_PBS) for an hour at 37 °C. After an hour, the wells were then washed once with PBS-T (0.05% (*v/v*) Tween 20 in PBS) buffer and rinsed once with PBS buffer. Sera from naïve and vaccinated mice were analysed (100 µL/well). All sera were first diluted ten-fold in 0.5% (*w/v*) BSA in PBS-T (0.05% (*v/v*) Tween 20 in PBS) buffer (BSA_5_PBS) and then prepared in serial two-fold dilutions. The diluted samples were added to the plate in triplicate and incubated for an hour at 37 °C. After an hour, the wells within the plate were washed twice with PBS buffer. An aliquot of 100 µL (1:2000 dilution) of horseradish peroxidase (HRP)-conjugated goat anti-mouse IgG (Invitrogen, Carlsbad, CA, USA) diluted in BSA_5_PBS-T was added and incubated for an hour at 37 °C. Any unbound IgG was removed by washing the plate twice with PBS-T buffer and then once with PBS buffer. After washing, the wells within the plate were stained with 100 µL of 3,3′,5,5′-Tetramethylbenzidine (TMB) substrate solution (SeraCare Life Sciences Inc, Milford, MA, USA) and incubated for 15 min at room temperature in the dark. The enzymatic reaction was stopped with 2 M sulphuric acid (H_2_SO_4_) (100 µL/well). Absorbance at 450 nm was measured using an Infinite M200 PRO microplate reader (TECAN, Männedorf, Switzerland).

### 2.10. Focus Reduction Neutralization Test (FRNT)

The neutralizing antibody titers were determined by FRNT based on the method described by Leng et al. (2009), with slight modifications [29]. Post-immunized sera were heat-inactivated at 56 °C for 30 min and diluted ten-fold in 1X DMEM. Serial two-fold dilutions of the inactivated sera were prepared and mixed with an equal volume of virus suspension containing 200 FFU (focus forming units) per well. Following incubation at 37 °C for 2 h, the virus-antibody mixture was then added in duplicate to the monolayers of confluent Vero cells. The infected cells were incubated at 37 °C in an incubator supplemented with 5% CO_2_ for 2 h to allow the virus to absorb to the cells. The media in the wells were then aspirated and replaced with 1.5% (*w/v*) carboxymethylcellulose (CMC) medium (Sigma-Aldrich, St Louis, MO, USA) containing 2% FBS and incubated for 4 days at 37 °C.

After incubation, the cells were fixed for 40 min at −20 °C with methanol:acetone (1:1). The fixed cells were treated with monoclonal antibody 4G2 for an hour. After incubation and washing with PBS, antibody-labeled cells were detected using a secondary antibody, the HRP-conjugated rabbit anti-mouse IgG (Sigma-Aldrich, St. Louis, MO, USA), for an hour. The labeling was visualized using TMB (SeraCare Life Sciences Inc, Milford, MA, USA). The neutralizing antibody titer was calculated as the reciprocal of the highest serum dilution that produced a 50% reduction of 200 FFU compared to the positive control well containing the virus and pre-immunized sera. Therefore, the FRNT_50_ titer was calculated by counting foci and reporting the titer as the reciprocal of the last serum dilution to show a 50% reduction of the foci count based on the number of foci from the positive control.

### 2.11. IFN-γ Enzyme-Linked Immunospot (ELISPOT) Assay

Activated antigen-specific T cell responses were evaluated using mice splenocytes harvested from BALB/c mice upon completion of the immunization protocol and the ELISPOT kit, according to the manufacturer’s instruction (Mabtech, Nacka Strand, Sweden). The 96-well filtration plates (Mabtech, Nacka Strand, Sweden) were pre-coated with anti-mouse IFN-γ capture antibody. The wells were washed and blocked with RPMI 1640 containing 10% FBS and 1% Pen-Strep. Splenocytes were added to the wells at 1 × 10^6^ cells/well. Peptides P1–P4 were used to stimulate the cells at the concentration of 10 µg/mL in a volume of 100 µL. Wells containing splenocyte cells were stimulated with 5.0 µg/mL phytohemagglutinin-L (PHA-L) (Sigma-Aldrich, USA), which served as the positive control. Wells containing unstimulated cells served as the negative control. Wells containing RPMI 1640 alone served as the background control. The plates were incubated at 37 °C in an incubator supplemented with 5% CO_2_ for 20 h. After incubation, the cells were removed and the plate was washed five times with PBS. The plate was then incubated with the detection antibody (biotinylated detector antibody, Mabtech, Nacka Strand, Sweden) for 2 h at room temperature. After incubation, unbound detection antibody was removed by washing and the enzyme conjugate (Streptavidin-HRP) was then added. After an hour of incubation at room temperature, the unbound enzyme conjugate was removed by washing and the plate was stained with a TMB substrate solution for 20 min. The plate was washed extensively with deionized water, and allowed to air-dry overnight. Spots were enumerated using an ELISPOT reader. Positive IFN-γ ELISpot responses were determined from spot forming cells (SFC)/well using a cutoff of at least 20 spots and a four-fold higher number of SFC versus the negative control.

### 2.12. Statistical Analysis

All values are expressed as the mean value ± standard deviation (SD). Significant differences between groups were analysed by one-way ANOVA analysis with Tukey’s multiple comparison test using Graph Pad Prism v8.02 software (Graph Pad Software Inc., San Diego, CA, USA). Differences were considered statistically significant at *p* < 0.05.

## 3. Results

### 3.1. Polystyrene-Based Nanovaccine Formulations

We designed four multi-epitope peptide vaccines by linking a universal T-helper epitope (PADRE or TpD) to the highly conserved CD8 T cell and B cell epitopes (B1 or B2) against the four DENV serotypes. These four multi-epitope peptides (P1–P4) were conjugated to 50 nm carboxylated PSNPs to generate the nanovaccine formulations (NP1–NP4). Using an optimized covalent conjugation protocol, all five peptides were conjugated to the PSNPs with an optimal efficiency of over 70%. The optimized conjugation conditions for each peptide are shown in Table 1.

Conjugations of peptides to PSNPs were also assessed visually using the TEM. Peptides were covalently attached to the surface of the PSNPs, but some partial peptide-coated PSNPs were observed in the nanovaccines. This resulted in a lower conjugation efficiency of between 73% and 76.7%.

### 3.2. Humoral Immune Responses

The IgG antibody titers were measured after the immunization of BALB/c mice via the subcutaneous route below the loose skin at the back of the neck with P1–P4 peptides and peptides conjugated to PSNPs (NP1–NP4). The sera were first collected from the vaccinated mice and mice from the control group at days 0, 14, 28, and 42, and the sera were serially diluted and tested using ELISA for IgG antibody responses. The highest levels of IgG antibodies were detected at day 42 (Table 2). Low levels of IgG antibodies (<80) were observed after mice were immunized with peptides P1–P4. In contrast, higher levels of IgG antibodies (<1280 to <10,240) were observed after mice were immunized with the nanovaccines (NP1–NP4), indicating that conjugations to PSNPs enhanced the IgG production and significant differences were observed when compared with the mice immunized with the PBS control (<10). The differences between the total IgG antibody titers induced by the immunization of mice with nanovaccines against the mice immunized with peptides alone were significant. The titers of IgG antibodies in mice immunized with nanovaccines (NP1–NP4) were higher than in mice immunized with only the multi-epitope peptide formulations: NP1 against P1 (<10,240 vs. <80, *p* < 0.0001), NP2 against P2 (<5120 vs. <80, *p* <0.0001), NP3 against P3 (<2560 vs. <80, *p* < 0.01), and NP4 against P4 (<1280 vs. <80, *p* < 0.05). The NP1 formulation was able to elicit the highest production of IgG titers (<10,240), followed by NP2 and NP3 formulations (NP2: <5120, NP3: <2560), with the NP4 formulation showing the least IgG antibody titers (<1280) (Table 2).

### 3.3. Neutralizing Antibody Responses

Sera at various dilutions were used to test the neutralizing activity against DENV1, DENV2, DENV3, and DENV4 in FRNT assays using Vero cells. The end-point titer was determined as the serum dilution causing a 50% reduction in the number of foci (FRNT_50_) when compared to the positive control containing the virus and infected Vero cells treated with the pre-immunized sera [30,31]. Mice immunized with the four multi-epitope peptide (P1–P4) vaccines showed low titers of neutralizing antibodies in the sera.

**Figure 2:**
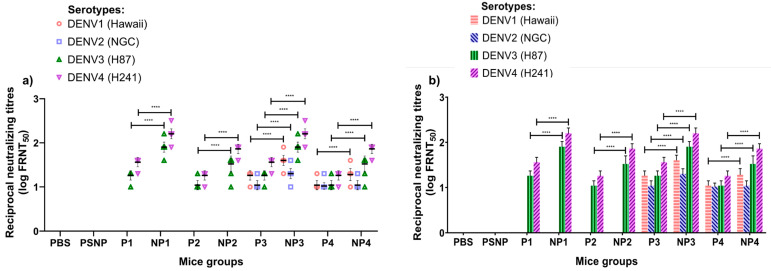
Neutralizing activity of anti-peptide antibodies against four dengue virus (DENV) serotypes. (**a**) Each dot represents the reciprocal antibody titer (log FRNT_50_) from an individual mouse in each group. (**b**) The average of reciprocal antibody titers (log FRNT_50_) in each mouse group. Data from triplicate assays were plotted using Graph Pad Prism Version 8.02 (Graph Pad Software Inc., San Diego, CA, USA) and expressed as the mean and SDs (one-way ANOVA analysis with Tukey’s multiple comparison test: **** *p* < 0.0001).

High levels of neutralizing activities were observed in the sera of mice immunized subcutaneously below the loose skin at the back of the neck with the NP1–NP4 nanovaccines when compared with the mice immunized with multi-epitope peptides (P1–P4). Among the four multi-epitope peptide formulations, P3 and P4 demonstrated low levels of neutralizing activities against all four DENV serotypes. In the mice vaccinated with nanovaccines, NP3 and NP4 demonstrated significantly higher levels of neutralizing activities against all four DENV serotypes, and NP3 was found to elicit higher neutralizing antibody titers when compared to NP4. Neutralizing antibodies against DENV serotypes 1 and 2 were undetectable when mice were immunized with either multi-epitope peptide vaccines (P1 and P2) or NP1 and NP2 nanovaccines. In contrast to this finding, mice immunized with multi-epitope peptide P3 and P4 vaccines showed low levels of neutralizing antibodies to DENV serotypes 1–4. However, the sera from mice immunized with the NP3 nanovaccine showed a more significant increase in neutralizing antibodies against all four serotypes. There was a two-fold increase in neutralizing antibodies against serotype 2 elicited by the NP3 nanovaccine when compared with the P3 peptide vaccine. The NP4 nanovaccine also elicited neutralizing antibodies to all four DENV serotypes, albeit at lower levels compared to NP3. The neutralizing antibody levels for each mouse are presented in Figure 2a.

Nanovaccines carrying P3 and P4 peptides shared the same CD4 T cell epitope (TpD), but different B cell epitopes (B1 or B2). The data indicated that the B1 epitope derived from the E protein was more effective than the B2 epitope derived from the NS4A protein in terms of the ability to induce higher neutralizing activities. The same observation was made with the higher levels of neutralizing antibodies against DENV serotypes 3 and 4 elicited by NP1 which contained the B1 peptide. However, no neutralizing antibodies were elicited against DENV1 and 2 serotypes upon immunization with NP1. The only difference between NP1 and NP3 was the presence of the universal TpD in NP3, whilst NP1 carried the PADRE CD4 T-helper epitope (Figure 2b).

### 3.4. IFN-γ Responses

**Figure 3:**
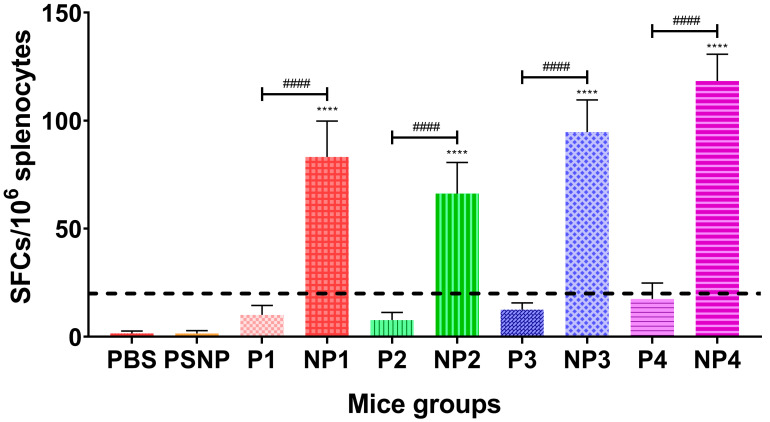
IFN-γ responses induced by multi-epitope peptide vaccines and nanovaccines. Data from triplicate assays were plotted using Graph Pad Prism Version 8.02 (Graph Pad Software Inc., San Diego, CA, USA) and expressed as the mean number of net spot forming cells (SFC) per 10^6^ splenocytes and SDs. Semi-dotted line indicates the minimal level of a positive response (net SFC per 10^6^ cells of ≥20). One-way ANOVA analysis with Tukey’s multiple comparison test: **** *p* < 0.0001 vs. PBS and #### *p* < 0.0001 vs. respective peptide.

On day 42, splenocytes were harvested and subjected to the IFN-γ ELISPOT assays using each of the peptides (P1–P4) as the stimulant. No significant IFN-γ was present in the control groups of mice (*n* = 5/group) inoculated with PBS or PSNP only. Mice immunized with P1–P4 peptides showed splenocytes secreting IFN-γ, but the induction of the IFN-γ response was poor for mice inoculated with only P1–P4 peptides (<20 SFC per 10^6^ splenocytes). An antigen-specific response could be considered as positive only when the net SFC ≥ 20 per 10^6^ cells [32]. Among the splenocytes inoculated with the multi-epitope peptide formulations, P4 was able to elicit an IFN-γ response of 18 ± 7 SFC per 10^6^ splenocytes, which was higher than those elicited by P3 (13 ± 3 SFC per 10^6^ splenocytes) and P1 (10 ± 4 SFC per 10^6^ splenocytes), whilst the P2 formulation gave the lowest IFN-γ response (8 ± 4 SFC per 10^6^ splenocytes) (Figure 3).

However, when mice were immunized subcutaneously below the loose skin at the back of the neck with the multi-epitope peptides conjugated to PSNPs, significant levels (six-fold higher levels) of IFN-γ were elicited when compared to vaccination with only peptides. These results showed that when peptides were covalently conjugated to PSNPs and presented as nanovaccines, potent cellular immune responses were induced.

NP4 was able to elicit the highest IFN-γ response (118 ± 12 SFC per 10^6^ splenocytes), followed by NP3 (95 ± 15 SFC per 10^6^ splenocytes). These IFN-γ levels were higher than those found in splenocytes from mice vaccinated with NP1 (83 ± 17 SFC per 10^6^ splenocytes) and NP2 (66 ± 14 SFC per 10^6^ splenocytes) formulations. Since NP3 and NP4 shared the same T-helper epitope (TpD) in the nanovaccine and elicited higher IFN-γ responses than NP1 and NP2, the data indicated that the TpD epitope was more effective than the PADRE epitope in terms of eliciting a higher IFN-γ response (Figure 3).

## 4. Discussion

DENV infection is a major public health problem in tropical and subtropical regions of the world. It has become the leading cause of hospitalizations and death among adults and children in some dengue-endemic Asian and Latin American countries [33]. Vaccines are urgently needed to prevent dengue and Dengvaxia^®^, an LAV, is currently the only vaccine licensed for human use. However, it can only be administered to vaccinees who have had prior exposure to DENV (seropositive individuals) [34]. Epidemiological data have strongly suggested that Dengvaxia^®^ sensitizes dengue-naïve vaccinees to ADE upon subsequent DENV infections [35].

Epitope-based peptide vaccines are free from live viruses and only contain selected B and T cell epitopes representing minimal antigenic epitopes which can be presented by MHC molecules to CD4^+^ and CD8^+^ T cells, thus enabling the induction of highly targeted immune responses [14]. The production of this type of vaccine is easy, fast, and cost-effective. It can be developed relatively quickly using an *in silico* approach to rationally design the vaccine [36]. However, the efficacy of the peptide epitopes predicted in silico requires validation in vivo [37].

The choice and selection of B and T cell epitopes are of the utmost importance in influencing the outcome of the vaccine efficacy. Bioinformatic tools can be helpful for predicting immunogenic epitopes such as B and T cell epitopes. In this study, B cell epitopes were obtained from two different DENV proteins (E and NS4A) based on *in silico* predictions. The B cell epitope B1 was suggested to be an immunodominant antigenic region conserved in all dengue serotypes based on the Immune Epitope Database (IEDB) [24,38]. This peptide is present in the fusion loop in domain II (DII) of the DENV E protein and is highly conserved in all four DENV serotypes [39]. The B cell epitope B2 is also highly conserved in all four DENV serotypes and was derived from the NS4A protein, which has been shown to induce B cell antibody responses in DENV-infected individuals [25,40].

The higher levels of IgG and neutralizing antibody (FRNT_50_) titers elicited in the murine model after immunization with PSNPs conjugated with the multi-epitope DENV peptide vaccine containing B1 derived from the E protein showed that it was more effective when compared to B2, which was derived from the NS4A protein. This observation supported the conclusion from previous studies stating that neutralizing antibodies were largely elicited by the E protein [41,42]. Li et al. (2011) evaluated a B cell epitope from the domain III (DIII) of the E protein of DENV2 [21]. However, no information was available to indicate the neutralizing capability of antisera against DENV1, DENV3, and DENV4. A serotype-specific neutralizing antibody response to only DENV2 would limit the protection conferred by the vaccine if dengue infection was caused by any of the other three serotypes. In endemic areas, it is very common to see a change of DENV serotype from one year to the next. Based on bioinformatic data from Mazumder et al. (2007), three peptides identified as conserved from the domain I (DI) (Pep01) and DII (Pep02 and Pep03) of the DENV E protein were evaluated [43,44]. The three peptides were able to induce a humoral response characterized by high levels of anti-peptide antibodies, but low levels of neutralizing antibodies were produced. Of the three epitopes, Pep03 was more immunogenic and it was able to elicit low levels of neutralizing antibodies against DENV1 and DENV3. As the neutralizing antibody levels were low (<20), the investigation concluded that further selection of the peptide sequence would be necessary to improve the production of higher levels of neutralizing antibodies and improve the immunogenicity of the peptide antigens [44]. In our study, NP1 and NP2 nanovaccines were not able to induce any neutralizing antibodies against DENV1 and DENV2, but NP3 was capable of eliciting neutralizing antibodies to all four serotypes, although the neutralizing antibodies against serotypes 1 (<40) and 2 (<20) were low compared to those elicited against serotypes 3 (<80) and 4 (<160).

Being highly conserved is not the only factor required for a peptide vaccine to be effective. The capability of the peptide to bind to multiple HLA-alleles could play a key role in immunogenicity. Major histocompatibility complex (MHC) class II molecules are encoded in humans by the HLA-DP, -DQ, and -DR genes and play a role in activating CD4^+^ T cells by presenting processed peptide antigens. The activated CD4^+^ T cells then undergo clonal expansion, prime CD8^+^ T cells, and drive the humoral response, assisting in B cell maturation and proliferation and initiating an antigen-specific IgG antibody response [45]. The incorporation of a universal CD4 T-helper epitope in a multi-epitope peptide vaccine can be beneficial in immune response enhancement. In the current study, two such universal T-helpers were incorporated—PADRE and TpD.

PADRE’s capability to bind to the majority of MHC class II alleles, with a high affinity to 15 of the 16 most common human HLA-DR types and moderate-to-high affinity to mouse I-A^b/d^ and I-E^b/d^ haplotypes, enables the epitope to overcome problems caused by the polymorphism of HLA-DR molecules in the population [26,46,47]. This epitope has demonstrated its ability to enhance the immune response of a peptide-based vaccine and it has also been employed in clinical trials with a minimal toxicity [28]. PADRE has been used in the development of a multi-epitope DENV peptide vaccine constructed by Li et al. (2011) [21]. TpD provides a broad MHC class II coverage in humans, having a high binding affinity primarily across HLA-DRB1, with some binding affinity to HLA-DRB3, -DRB4, -DRB5, DP, and DQ alleles, and it is potent in enhancing long-term CD4 immune responses in mice, non-human primates, and humans [20]. In addition to enhancing antigen-specific IgG antibodies, inclusion of the TpD epitope has also demonstrated enhanced neutralization activity in vitro. It is considered a valuable epitope for eliciting enhanced responses against poorly immunogenic vaccines [20,48].

In the current study, both universal T-helper epitopes were equally effective in terms of IgG production. However in the context of neutralization activity, P3 and P4 peptides, which shared the TpD T-helper epitope, were capable of eliciting neutralizing antibodies against all four serotypes, whereas P1 and P2 could only elicit neutralizing antibodies against DENV3 and DENV4. The levels of neutralizing antibody titers were then enhanced when P3 and P4 peptides were conjugated to PSNPs. NP1 and NP3 were observed to have differences in their neutralizing antibody response, despite sharing the same B1 epitope derived from the E protein, suggesting that the T-helper epitope may play a role in this. The herpes simplex virus type 2 vaccine developed by Li et al. (2018) was adjuvanted with TpD, which successfully increased the antigen-specific response and was reported to enhance the neutralizing activity in both the serum and mucosal surface, as well as induce long-term protective immunity [48].

In addition to the humoral response, cellular immune responses are also important in limiting and clearing viral infections. Incorporating a highly conserved and high HLA coverage are the common approaches employed in designing an effective multi-epitope peptide vaccine. A CD8^+^ T cell epitope with the capacity to bind directly to multiple HLA alleles would be ideal to overcome the need for individualized vaccinations [18]. The CD8^+^ T cell epitope incorporated in the multi-epitope peptide construct in this study was derived from the NS5 protein. This highly conserved epitope was identified by *in silico* prediction by Shi et al. (2015) and exhibited a 47.16% population coverage in China, binding to some HLA-B and HLA-C alleles [27].

In this study, nanovaccines (NP1–NP4) resulted in a significant improvement in the production of total IgGs, neutralizing antibodies, and IFN-γ responses, demonstrating a self-adjuvanting capability without the need to use conventional adjuvants, such as Freund’s adjuvant, in the immunization of mice. Previous studies with mice and sheep antigens covalently conjugated to non-inflammatory biocompatible PSNPs (40–50 nm) showed their abilities to induce potent CD4^+^ and CD8^+^ T cell responses, as well as antibody responses, in vivo [22,49]. Based on multiple studies, these PSNP-based vaccines coated with the target antigen of choice have demonstrated their ability to promote potent immunological responses and avoid concomitant inflammatory responses at the site of injection. PSNPs are inert and biocompatible, and they have repeatedly been shown to be safe and well-tolerated in many animal models at both low and high doses [49,50,51]. In this study, IFN-γ responses were low in the mice vaccinated with only multi-epitope peptide vaccines, but they were significantly improved when the peptides were conjugated with PSNPs. It is unknown at this stage whether extrinsic adjuvants such as toll-like receptor (TLR) or dendritic cells targeting ligands could further enhance the immunogenicity of PSNP-conjugated multi-epitope peptide vaccines [23,49,50,52].

The development and use of a monovalent epitope-based peptide vaccine against a single serotype such as DENV2 might not be advisable, as there is no protection against heterologous serotypes that might subsequently infect the same host [21]. In addition, as the serotype-specific neutralizing antibodies might wane over a period of time, the risk of ADE that could lead to severe dengue is challenging. The titers of neutralizing antibodies against serotypes 3 and 4 (1:80–1:160) elicited by the nanovaccine NP3 were within the range (>1:80–1:320) that has been shown to confer protective effects in humans [53]. Neutralizing titers elicited by NP3 against serotypes 3 and 4 were significantly higher than the neutralizing titer reported by Li et al. against serotype 2 (1:64). Despite the lower level of neutralizing titer observed, the PADRE linked to A1 and A2 peptides presented in the P1 vaccine developed by Li et al. (2011) was able to confer the protection of mice by reducing viremia [21].

Alternatively, epitopes that are capable of eliciting high levels of neutralizing antibodies against DENV1 and DENV2 could be incorporated into the NP3 nanovaccine. For example, the linked A1 and A2 peptide reported to elicit both Th1 and Th2 responses against DENV2 could be incorporated into the NP3 nanovaccine. This may lead to the ability of NP3 to generate higher levels of neutralizing antibodies and cellular immune responses against all four DENV serotypes and confer cross-protection. Besides, the addition of CpG or the incorporation of other linked-molecular adjuvants (TLR or NOD-like receptor (NLR)) in NP3 could also potentially improve the immunogenicity of the NP3 nanovaccine [54,55,56]. The efficacy of the NP3 nanovaccine as a tetravalent dengue vaccine requires further exploration in future challenge studies to assess its ability to protect mice from dengue infections and more importantly, to evaluate its risk of inducing DENV-enhancing antibodies that could trigger the ADE phenomenon.

## 5. Conclusions

In conclusion, the carboxylated PSNPs used in this study demonstrated efficacy as a nanoadjuvant which could increase the immunogenicity of synthetic peptides, without the need for conventional toxic adjuvants. A significant humoral response was observed from the neutralizing antibody titers elicited by the multi-epitope peptide-based nanovaccines, especially NP3. It had neutralizing antibody levels that were significantly higher than those of previously published peptide-based vaccines, especially against serotypes 3 and 4. NP3, which contained the TpD linked to the B1 epitope, was demonstrated to be more effective in inducing neutralizing antibodies against all four DENV serotypes when compared to the nanovaccine NP1 carrying PADRE linked to the same B1 epitope. In addition, NP3 demonstrated the ability to elicit higher titers of neutralizing antibodies against DENV serotypes 3 (1:80) and 4 (1:160) when compared to NP4 carrying the same TpD, but linked to a different B cell epitope (B2). Among the four nanovaccine constructs developed in this study, NP3 has the potential to be developed as a tetravalent dengue vaccine, but further studies involving specific B and T cell epitopes or adjuvants are required to improve the humoral and cellular responses.

## Figures and Tables

**Table 1:** Optimized conjugation conditions, efficiency, and size of the nanovaccine formulations.

Nanovaccines ^a^	Conjugation pH ^b^	Conjugation Efficiency (%) ^c^	Recovery of Nanoparticles (%)	Size (nm)
**NP1**	6.5	76.0 ± 2.6	88.3 ± 1.5	98.1 ± 1.9
**NP2**	6.5	76.7 ± 2.1	87.0 ± 1.1	100.8 ± 2.9
**NP3**	7.5	75.3 ± 2.2	90.3 ± 1.5	63.1 ± 1.1
**NP4**	7.5	73.0 ± 2.4	88.0 ± 1.6	63.4 ± 1.4

^a^: NP1–NP4: P1–P4 conjugated to polystyrene nanoparticles (PSNPs). ^b^: Conjugation pH refers to the pH condition of the 2-*N*-morpholino-ethanesulfonic acid (MES) buffer used for incubation during the peptide and carboxylated PSNPs mixture. ^c^: Conjugation efficiency was determined as the percentage of peptide antigen successfully conjugated to PSNPs.

**Table 2:** Time course of IgG antibody production elicited by the multi-epitope constructs containing the respective peptides in immunized mice.

Mice Groups	Mean Reciprocal IgG Antibody Titer			
	Day 0	Day 14	Day 28	Day 42
PBS	<10	<10	<10	<10
PSNP	<10	<20	<40	<40
P1	<10	<20	<40	<80
NP1	<10	<80	<2560	<10,240
P2	<10	<20	<40	<80
NP2	<10	<80	<1280	<5120
P3	<10	<20	<40	<80
NP3	<10	<40	<320	<2560
P4	<10	<20	<40	<80
NP4	<10	<40	<160	<1280

The IgG titers were determined by indirect ELISA using peptides as the capture antigen.
